# Validation of a Case Definition to Identify Patients Diagnosed With Cardiovascular Disease in Canadian Primary Care Practices

**DOI:** 10.1016/j.cjco.2023.04.003

**Published:** 2023-04-22

**Authors:** Riddhima Dinah Thomas, Leanne Kosowan, Mary Rabey, Alan Bell, Kim A. Connelly, Nathaniel M. Hawkins, Carolyn Gall Casey, Alexander G. Singer

**Affiliations:** a UCD School of Medicine, University College Dublin, Belfield, Dublin, Ireland; b Department of Family Medicine, Rady Faculty of Health Sciences University of Manitoba, Winnipeg, Manitoba, Canada; c Faculty of Medicine, University of Limerick, Limerick, Ireland; d Department of Family and Community Medicine, University of Toronto, Toronto, Ontario, Canada; e Keenan Research Centre for Biomedical Science, Unity Health, St. Michael’s Hospital, University of Toronto, Toronto, Ontario, Canada; f Centre for Cardiovascular Innovation, Division of Cardiology, University of British Columbia, Vancouver, British Columbia, Canada; g Canadian Cardiovascular Society, Ottawa, Ontario, Canada

## Abstract

**Background:**

Cardiovascular disease (CVD) is a leading cause of death globally. This study validates a primary care-based electronic medical record case definition for CVD.

**Methods:**

This retrospective, cross-sectional study explores electronic medical record data from 1574 primary care providers participating in the Canadian Primary Care Sentinel Surveillance Network. A reference standard was created by reviewing medical records of a subset of patients in this network (n = 2017) for coronary artery disease (CAD), cerebrovascular disease (CeVD), and peripheral vascular disease (PVD). Together, these data produced a CVD reference. We applied validated case definitions to an active patient population (≥ 1 visit between January 1, 2018 and December 31, 2019) to estimate prevalence using the exact binomial test (N = 689,301). Descriptive statistics, χ^2^ tests, and *t* tests characterized patients with vs without CVD.

**Results:**

The optimal CVD Case Definition 2 had a sensitivity of 68.5% (95% Confidence Interval [CI]: 61.6%-74.8%), a specificity of 97.8% (95% CI: 97.0%-98.4%), a positive predictive value of 77.7% (95% CI: 71.6%-82.7%), and a negative predictive value of 96.5% (95% CI: 95.8%-97.1%). Included in this CVD definition was a strong CAD case definition with sensitivity of 91.6% (95% CI: 84.6%-96.1%), specificity of 98.3% (95% CI: 97.6%-98.8%), a PPV of 74.8% (95% CI: 67.8%-80.7%), and an NPV of 99.5% (95% CI: 99.1%-99.7%). This CVD definition also included CeVD and PVD case definitions with low sensitivity (77.6% and 36.6%) but high specificity (98.6% and 99.0%). The estimated prevalence of CVD among primary care patients is 11.2% (95% CI, 11.1%-11.3%; n = 77,064); the majority had CAD (6.4%).

**Conclusions:**

This study validated a definition of CVD and its component parts—CAD, CeVD, and PVD. Understanding the prevalence and disease burden for patients with CVD within primary care settings can improve prevention and disease management.

## Graphical abstract

**Figure:**
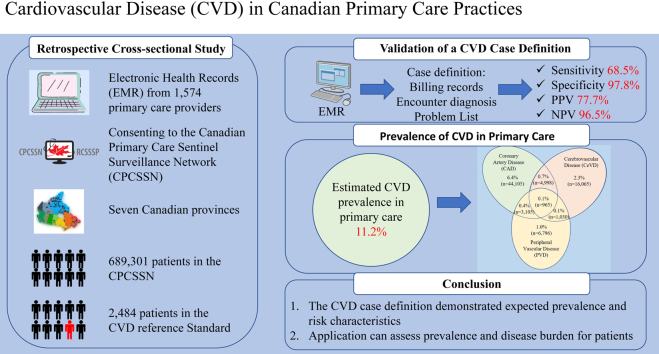

## Résumé

**Contexte:**

La maladie cardiovasculaire (MCV) est l’une des premières causes de décès dans le monde. Cette étude valide une définition de cas dans les dossiers médicaux électroniques en soins primaires pour la MCV.

**Méthodologie:**

Cette étude intersectionnelle rétrospective explore les données provenant des dossiers médicaux électroniques de 1 574 fournisseurs de soins primaires participant au Réseau canadien de surveillance sentinelle en soins primaires. Une norme de référence a été créée en vérifiant les dossiers médicaux d’un sous-ensemble de patients dans ce réseau (n = 2017) à la recherche d’une coronaropathie, d’une maladie cérébrovasculaire ou d’une maladie vasculaire périphérique. Ensemble, ces données ont produit une référence pour la MCV. Nous avons appliqué des définitions de cas validées à une population de patients actifs (≥ 1 consultation entre le 1^er^ janvier 2018 et le 31 décembre 2019) pour estimer la prévalence à l’aide du test binomial exact (N = 689 301). Des statistiques descriptives, des tests de χ^2^, et des tests *t* ont permis de caractériser les patients atteints ou non de MCV.

**Résultats:**

La définition de cas n^o^ 2 optimale pour la MCV avait une sensibilité de 68,5 % (intervalle de confiance [IC] à 95 % : de 61,6 à 74,8 %), une spécificité de 97,8 % (IC à 95 % : de 97,0 à 98,4 %), une valeur prédictive positive de 77,7 % (IC à 95 % : de 71,6 à 82,7 %) et une valeur prédictive négative de 96,5 % (IC à 95 % : de 95,8 à 97,1 %). On a inclus à cette définition de MCV une définition d’un cas de coronaropathie puissante, avec une sensibilité de 91,6 % (IC à 95 % : de 84,6 à 96,1 %), une spécificité de 98,3 % (IC à 95 % : de 97,6 à 98,8 %), une valeur prédictive positive de 74,8 % (IC à 95 % : de 67,8 à 80,7 %) et une valeur prédictive négative de 99,5 % (IC à 95 % : de 99,1 à 99,7 %). Cette définition de la MCV incluait aussi des cas de maladie cérébrovasculaire et de maladie vasculaire périphérique ayant une faible sensibilité (77,6 % et 36,6 %), mais une spécificité élevée (98,6 % et 99,0 %). La prévalence estimée de la MCV parmi les patients en soins primaires est de 11,2 % (IC à 95 % : de 11,1 à 11,3 %; n = 77 064); la majorité présentant une coronaropathie (6,4 %).

**Conclusions:**

Cette étude validait une définition de la MCV et de ses composantes – coronaropathie, maladie cérébrovasculaire et maladie vasculaire périphérique. Une compréhension de la prévalence et du fardeau de la maladie pour les patients atteints de MCV en soins primaires peut améliorer la prévention et la prise en charge de cette maladie.

Cardiovascular disease (CVD) refers to several conditions that affect blood vessels, including coronary artery disease (CAD), cerebrovascular disease (CeVD), and peripheral vascular disease (PVD).^1^, ^2^, ^3^, ^4^, ^5^, ^6^, ^7^, ^8^, ^9^ CVD contributes to significant direct and indirect healthcare costs. Globally, CVD is the leading cause of mortality and accounts for approximately 30% of all deaths.^1^^,^^2^^,^^5^ Most of the mortality associated with CVD relates to deaths from CAD and CeVD (ie, strokes).^1^^,^^2^^,^^5^, ^6^, ^7^, ^8^, ^9^, ^10^ A third of these deaths are considered premature, involving individuals under the age of 70 years.^1^^,^^2^^,^^7^

Underlying determinants of CVD are linked to not only hereditary factors but also the social determinants of health.^1^^,^^2^ Poverty, stress, urbanization, population aging, and globalization are all considered important drivers of CVD.^1^ Specifically, patient risk factors for CVD include obesity, comorbidities, health behaviours, and family history, as well as psychosocial factors.^3^, ^4^, ^5^, ^6^^,^^10^, ^11^, ^12^, ^13^, ^14^, ^15^, ^16^, ^17^, ^18^, ^19^, ^20^, ^21^ These risk factors often interact and amplify the risk of vascular damage leading to CVD morbidity and mortality of patients at younger ages.^11^^,^^12^ Canadian and international guidelines recommend broad implementation of measures in primary care settings to address CVD and related risk factors.^1^^,^^3^, ^4^, ^5^, ^6^^,^^11^^,^^12^ Identifying CVD within primary care can support improved prevention, treatment, and management, which in turn can prevent premature mortality.^1^^,^^3^, ^4^, ^5^, ^6^^,^^11^^,^^12^

Efforts have been made to utilize healthcare administrative databases for disease surveillance, but these efforts have not been performed consistently with primary care electronic medical record (EMR) data.^22^ Although EMR-based case definitions have been validated for several related conditions, CVD case definitions have been developed with largely administrative health data, including hospital records.^23^^,^^24^, ^25^, ^26^, ^27^, ^28^, ^29^, ^30^, ^31^, ^32^, ^33^, ^34^ CVD definitions based on administrative health data may lead to underreporting of some CVD presentations that precede hospitalization.^35^, ^36^, ^37^ The widespread adoption of EMRs has resulted in the availability of primary care EMR data, including information on diseases and their characteristics not currently captured in administrative data sources.^23^^,^^38^ Primary care EMR data can improve case detection, thereby providing insight into the epidemiology of diseases and informing prevention, management, and quality improvement strategies.^22^^,^^24^^,^^38^, ^39^, ^40^ EMR-based primary care prevalence estimates can measure efforts to reduce disease burden through identification of patients and an understanding of primary and secondary prevention and treatments.

Clinical notes, diagnoses, examination results, laboratory data, risk factors, and prescribing practices are all important components of EMR data that have enormous potential to be used to describe the care provided for conditions such as CVD.^23^ We sought to develop, validate, and apply an EMR-based case definition of CVD, with the goal of detecting and characterizing patients who have CVD, in primary care settings. Further, we characterize the prevalence of and overlap between the component parts of CVD.

## Materials and Methods

This retrospective cross-sectional study assessed EMR data held in the Canadian Primary Care Sentinel Surveillance Network (CPCSSN). On a semiannual basis, CPCSSN extracts and cleans deidentified longitudinal primary care EMR data from 7 Canadian provinces (British Columbia, Alberta, Manitoba, Ontario, Quebec, Nova Scotia, and Newfoundland and Labrador). Provincial networks extract EMR data from consenting family physicians, nurse practitioners, and community pediatricians, and transfer deidentified data to the CPCSSN to create a single pan-Canadian EMR-based data repository representing > 1,800,000 Canadians. Data are included for all patients who attended an appointment with a consenting provider, unless choose to optto not be included. The repository includes structured data fields and short-text fields, including billing, health condition(s) (problem list), encounter diagnoses, medication(s), and exam, plus patient and provider tables. A cohort was created of active patients (defined as those seen at least once in the past 2 years) from the CPCSSN repository, with data available up to December 31, 2019. The starting point of the data is dependent on when providers initiated their use of their EMR, with most records going back 10 or more years.

### Reference set

Among active patients from the CPCSSN repository, we created a subset of randomly selected patients’ EMR data for complete medical records review, to produce a positive and negative reference set for CVD. Random selection of records was performed using a random number generator within Microsoft Structured Query Language (SQL) Server that accounted for the province of residency. Medical students and family medicine residents reviewed 2 files, representing the EMRs for 2484 patients. The first was the clinical encounter note, which contains a description of each encounter, written by the provider, and may include information on diagnosis, medication, blood pressures, and laboratory ordering. The second file included free-text entries in the health conditions, billing, and encounter diagnosis table representing the diagnosis name typed into the EMR by the primary care provider following the patient’s encounter*.* Chart reviewers populated the data extraction table, which included the following columns: diagnosis name; CVD (yes/no); type of CVD (ie, CAD, CeVD, PVD); related conditions (eg, myocardial infarction, ST elevation myocardial infarction, stroke, vascular ulcer); and documented symptoms and medications (Supplemental Appendix S1).

**Figure 1:**
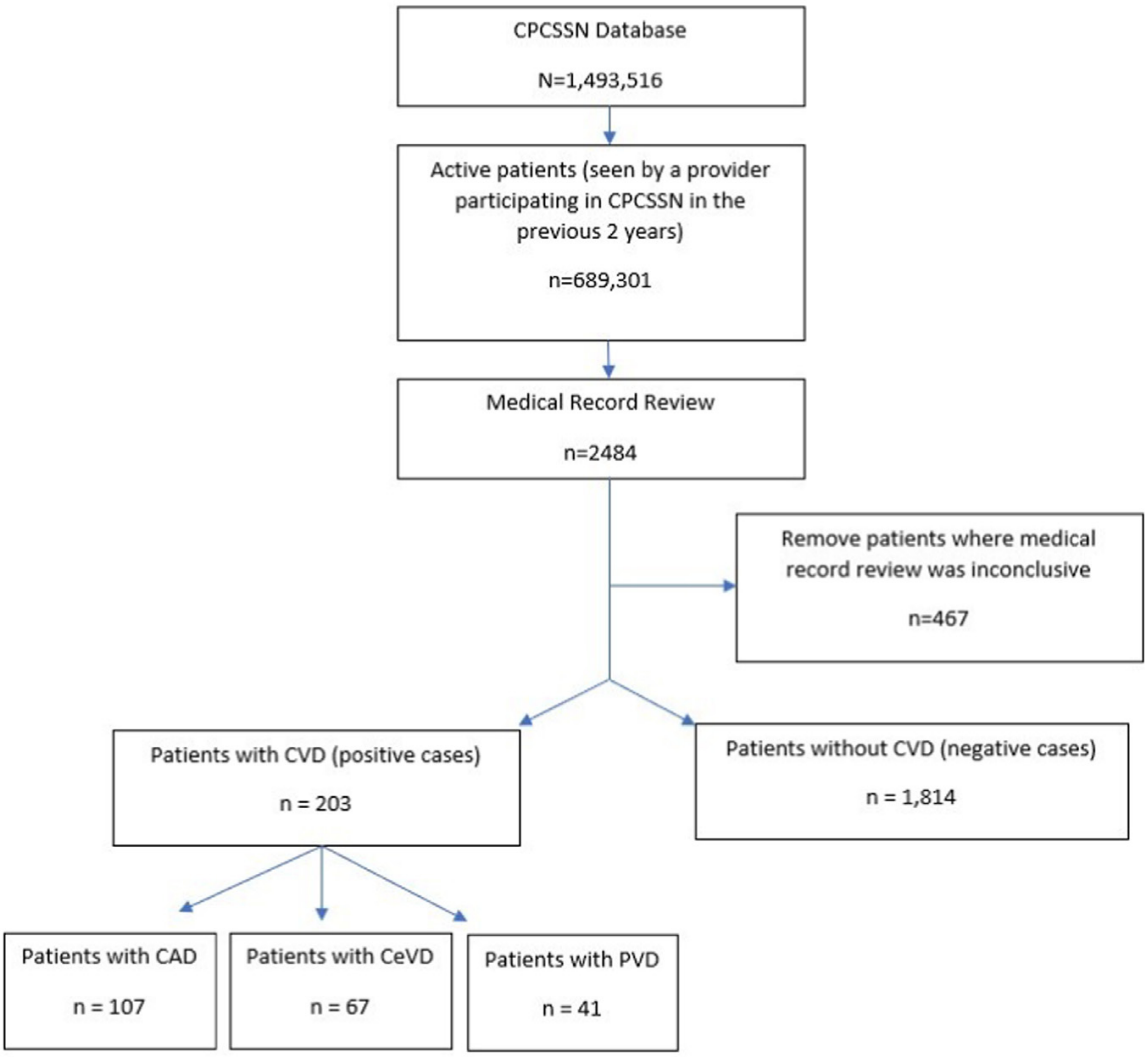
Flow diagram for creation of the cardiovascular disease (CVD) reference set from the Canadian Primary Care Sentinel Surveillance Network (CPCSSN). CAD, coronary artery disease; CeVD, cerebrovascular disease; PVD, peripheral vascular disease.

The Public Health Agency of Canada^7^^,^^8^ suggests that the Canadian prevalence of heart disease is 8%. Using an estimated prevalence of at least 8%, a margin of error of 5%, and a desired power of 80%, the sample size required is 201 positive cases. Our reference set has 203 positive cases and 1814 negative cases (Fig. 1). Patients within our positive reference set required that a CVD diagnosis (CVD, CAD, CeVD, or PVD) be documented in the EMR by their primary care provider. Some providers did not include a specific CVD diagnosis and documented “cardiovascular disease” as the diagnosis, whereas other providers included a specific diagnosis for CAD, CeVD, or PVD. Cases were reviewed further for the presence of symptoms, medications, and referrals, to demonstrate the validity of the diagnosis. Records were reviewed by ≥ 2 medical students/residents. Discrepancies were highlighted and reviewed by a third reviewer. Negative cases did not include any indication of CVD in the EMR, including in diagnosis, medications, blood pressures, laboratory ordering, or referrals.

### Case definitions

We reviewed the literature for previously applied CVD case definitions^22^^,^^28^, ^29^, ^30^, ^31^, ^32^, ^33^, ^34^, ^35^, ^36^, ^37^ and tested our reference set compared to case definitions for CVD, including separate definitions for CAD, CeVD, and PVD (Supplemental Appendix S2). Literature and clinical informed case definitions produced 20 case definitions for CAD, 5 case definitions for CeVD, and 2 case definitions for PVD (Supplemental Appendix S2). CVD Case Definitions 1 and 2 were created using CAD, CeVD, and PVD case definitions that had strong sensitivity, specificity, positive predictive value (PPV) and negative predictive value (NPV; Supplemental Appendix S2). CAD Case Definitions 15 and 18 were similar in their approach, so we chose to apply CAD Case Definitions 13 and 15 in the CVD Case Definitions 1 and 2, respectively. CVD Case Definitions 3 and 4 applied administrative-based CVD definitions (Table 1).^23^^,^^25^, ^26^, ^27^, ^28^, ^29^, ^30^, ^31^, ^32^, ^33^, ^34^ Our definition focused on purely the vascular aspects comprising CVD, and omitted risk factors (ie, hypertension) and other sequelae of cardiac disease (ie, heart failure, arrhythmias, and valvular diseases) that have been included in some administrative data-based definitions to capture more general cardiac disorders. Patients captured with CAD, CeVD, or PVD were considered a CVD-positive case. We identified prescribed medications using anatomical therapeutic chemical (ATC) codes (cardiac therapy [ATC codes: C01DA02, C01DA05, C01DA08, C01DA14, C01EB09]; beta-blockers [ATC code: C07]; calcium-channel blockers [ATC codes: C08CA01, C08CA02, C08DA01]; angiotensin-converting enzyme inhibitors [ATC codes: C09A, C09B]; and angiotensin receptor blocker (ATC codes: C09C, C09D]). We compared the level of agreement between our reference set and the CVD case definitions (Table 1).

**Table 1:** Cardiovascular disease case definitions for validation in the reference set

Case definition	
Case Definition 1	CAD: ≥ 1 health condition, billing or encounter dx for ICD-9 codes 410-414 AND ≥ 2 medications for ATC codes starting with C01, C07, C08, C09 **OR** CeVD: ≥ 1 health condition, billing or encounter dx for ICD-9 codes 430-438 **OR** PVD: ≥ 1 health condition, billing or encounter dx for ICD-9 codes 440.xx, or 443.xx
Case Definition 2	CAD: ≥ 1 health condition, billing or encounter dx for ICD-9 codes 410-414 **OR** CeVD: ≥ 1 health condition, billing or encounter dx for ICD-9 codes 430-438 **OR** PVD: ≥ 1 health condition, billing or encounter dx for ICD-9 codes 440.xx, or 443.xx
Case Definition 3	≥ 1 health condition, billing or encounter dx for ICD-9 codes 390-429, 430-448,458 **OR** ≥ 1 ATC code from medication table for B01A, C01A, C01B, C01CA17, C01D, C02AA, C02AB, C02C, C02D, C02L, C03, C04AD, C05BA, C07AA01, C07AA02 C07AA03, C07AA04, C07AA06, C07AA07, C07AA12, C07AB, C07AG, C07B, C07C, C08, C09, C10
Case Definition 4	≥ 1 health condition, billing or encounter dx within 1 year for ICD-9 codes 390-429, 430-448,458 **OR** ≥ 1 ATC code from medication table within 1 year for B01A, C01A, C01B, C01CA17, C01D, C02AA, C02AB, C02C, C02D, C02L, C03, C04AD, C05BA, C07AA01, C07AA02, C07AA03, C07AA04, C07AA06, C07AA07, C07AA12, C07AB, C07AG, C07B, C07C, C08, C09, C10

ATC, anatomical therapeutic chemical; CAD, coronary artery disease; CeVD, cerebrovascular disease; dx, diagnoses; ICD-9, International Classification of Diseases, ninth edition; Clinical Modification; PVD, peripheral vascular disease.

### Statistical analyses

We compared the agreement of our reference set to case definitions using a 2 × 2 contingency table. We assessed the following metrics: sensitivity, specificity, PPV, NPV, and overall accuracy for CVD, CAD, CeVD, and PVD case definitions. The equations for these metrics are as follows:

PPV=TP/(TP+FP);

sensitivity=TP/(TP+FN);

NPV=TN/(TN+FN);

specificity=TN/(TN+FP);and

accuracy=(TP+TN)/(TP+FP+FN+TN),

where TP = number of true positive; TN = number of true negative; FN = number of false negative; and FP = number of false positive:

We used the exact binomial test to estimate prevalence and respective confidence intervals. We evaluated the CAD, CeVD, and PVD definitions using sensitivity, specificity, PPV, and NPV to determine the best-performing case definition. Using the best-performing definition, we describe patients with vs without CVD, using descriptive statistics, including mean, standard deviation (SD), and frequency. We explore differences in patients with vs without CVD using χ^2^ and *t* tests. Patient age was calculated at the index date of December 31, 2019. We applied CPCSSN validated case definitions for hypertension and diabetes.^38^ We identified conditions of interest using the following International Classification of Diseases, 9th revision (ICD-9) codes: dyslipidemia (ICD-9 code starting with 272); and heart failure (HF)^40^ (ICD-9 codes starting with 428 or 425 or angiotensin-converting enzyme/ angiotensin receptor blocker and beta-blocker and diuretic prescribed). We used the most recent body mass index and blood pressure recorded in the EMR for each patient. A body mass index ≥ 30 was considered obese. Visit frequency is an average of the mean number of visits in the preceding 3 years (2017, 2018, 2019).

Statistical analyses were conducted using SAS version 9.4 (SAS Institute, Cary, NC).

This study was approved by the Health Research Ethics Board at the University of Manitoba. The research reported in this paper adhered to the Strengthening the Reporting of Observational Studies in Epidemiology (STROBE) checklist for cross-sectional studies.

## Results

Our reference set included 2017 patients, with 203 positive cases and 1814 negative cases (Fig. 1). Among the positive cases were 107 patients who had CAD, 67 patients who had CeVD, and 41 patients who had PVD (Fig. 1). Patients may have been labelled as having multiple components of CVD. A total of 15 patients had CAD and CeVD, 11 patients had CAD and PVD, < 5 patients had CeVD and PVD, and < 5 patients had CAD, CeVD, and PVD. In 20 cases, documentation indicated “cardiovascular disease” but without sufficient detail to determine the type of CVD. A total of 467 patients were excluded because medical record review was inconclusive and reviewers could not be certain if the patient did or did not have CVD. Using the kappa statistic, the inter-rater agreement was 96.7% (95% Confidence Interval [CI] 95.1%-98.4%). Our reference set included patients from the following Canadian provinces: Alberta (11.5%); Ontario (25.7%); Quebec (0.6%); Newfoundland and Labrador (0.2%); Manitoba (58.2%); Nova Scotia (0.1%); and British Columbia (3.7%). Representation in each province was influenced by the number of providers participating in the CPCSSN and the availability of free-text encounter notes within the repository. Patients within the reference set had a mean age of 57 years (compared to 42 years in the Canadian Census) and were more likely to be female (57.7% vs 50.7%).^41^

Four case definitions for CVD were used. CVD Case Definitions 1 and 2 were developed based on case definitions validated to capture CAD, CeVD, and PVD. Case Definition 2 had the strongest agreement with our reference set. CVD Case Definition 2 was built using the best-performing CAD, CeVD, and PVD case definitions (ie, Case Definition 15 for CAD, and Case Definition 1 for CeVD and PVD) and had a sensitivity of 76.9% (95% CI 70.4%-82.5%), a specificity of 97.2% (95% CI 96.3%-97.9%), a PPV of 75.4% (95% CI 69.8%-80.2%), and an NPV of 97.4% (96.7%-97.8%). CVD Case Definition 1 was similar to Case Definition 2 but used CAD Case Definition 13, which requires ≥ 2 prescriptions for a related medication, thereby reducing the sensitivity to 68.5% (95% CI 61.6%-74.8%; Table 2)

**Table 2:** Agreement between National Reference Set for cardiovascular disease, and cardiovascular disease case definitions (N = 2017)

Case definition	Sensitivity, %(95% CI)	Specificity, %(95% CI)	Positive predictive value, % (95% CI)	Negative predictive value, % (95% CI)	Accuracy, %(95% CI)
Case Definition 1	68.47 (61.6–74.8)	97.79 (97.01–98.42)	77.65 (71.61–82.72)	96.52 (95.77–97.14)	94.84 (93.79–95.77)
Case Definition 2	76.85 (70.43–82.46)	97.19 (96.32–97.9)	75.36 (69.79–80.2)	97.4 (96.69–97.97)	95.14 (94.11–96.04)
Case Definition 3	94.58 (90.51–97.26)	60.09 (57.79–62.35)	20.96 (19.9–22.06)	99.0 (98.24–99.44)	63.56 (61.42–65.66)
Case Definition 4	59.11 (52.01–65.94)	78.17 (76.2–80.05)	23.26 (20.79–25.92)	94.47 (93.53–95.28)	76.25 (74.33–78.09)

More specifically, among the 20 case definitions for CAD (Supplemental Appendix S2), 3 had strong agreement with our CAD reference set. Case Definition 15 for CAD (ie. ≥ 1 ICD-9 codes 410-414 in billing/encounter diagnosis or health condition tables) had a sensitivity of 91.6% (95% CI 84.6%-96.1%), a specificity of 98.3% (95% CI 97.6%-98.8%), a PPV of 74.8% (95% CI 67.8%-80.7%), and an NPV of 99.5% (95% CI 99.1%-99.7%). This definition was similar to Case Definition 13 (ie, ≥ 1 ICD-9 codes 410-414 in billing/encounter diagnosis or health condition tables, and ≥ 2 CVD medications; sensitivity 72.0% [95% CI 62.5%-80.2%], specificity 99.1% [95% CI 98.6%-99.5%]), and Case Definition 17 (ie, ≥ 1 health condition for codes 410-414, OR ≥ 2 billing or encounter ICD-9 codes 410-414; sensitivity 79.4% [95% CI 70.5%-86.6%], specificity 98.9% [95% CI 98.3%-99.3%]).

Five definitions were tested for capturing CeVD (Supplemental Appendix S2). Two had agreement statistics around 70%. Case Definition 1 (ie, ≥ 1 ICD-9 codes 430-438 in billing/encounter diagnosis or health condition tables) had a sensitivity of 77.6% (95% CI 65.8%-86.9%), a specificity of 98.6% (95% CI 97.9%-99.0%), a PPV of 65.0% (95% CI 55.7%-73.3%), and an NPV of 99.2% (95% CI 98.8%-99.5%). Case Definition 5 (ie, ≥ 1 health condition for ICD-9 codes 430-438, OR ≥ 2 billing or encounter ICD-9 codes 430-438) had a sensitivity of 64.2% (95% CI 51.5%-75.5%), a specificity of 99.1% (95% CI 98.6%-99.5%), a PPV of 70.5% (95% CI 59.3%-79.6%), and an NPV of 98.8% (95% CI 98.3%-99.1%).

Two case definitions were tested for PVD—Case Definition 1 (ie, ≥ 1 ICD-9 codes 440.xx or 443, 443.9 in billing/encounter diagnosis or health condition tables; sensitivity 36.6% [95% CI 22.1%-53.1%], specificity 99.0% [95% CI 98.4%-99.4%], PPV 42.9% [95% CI 29.3%-57.6%], NPV 98.7% [95% CI 98.4%-99.0%]); and Case Definition 2 (≥ 2 ICD-9 codes 440.xx or 443, 443.9; sensitivity 12.2% [95% CI 4.1%-26.2%]), specificity 99.8% [95% CI 99.4%-99.9%], PPV 50.0% [95% CI 23.1%-76.9%], NPV 98.2% [95% CI 98.0%-98.4%]; Supplemental Appendix S2).

CVD Case Definitions 3 and 4 were based on administrative case definitions, which aimed to capture heart conditions more broadly and were inclusive of related illness and risk factors such as hypertension, atrial fibrillation, and HF. Due to these inclusion criteria, Case Definitions 3 and 4 had a much lower PPV (21.0%, 95% CI 19.9%-22.1%, and 23.3%, 95% CI 20.8%-25.9%, respectively; Table 2). However, assessment of the agreement of CVD Case Definitions 3 and 4 within a reference set of patients with CVD, hypertension, HF, or nonvalvular atrial fibrillation showed that our agreement had markedly improved. Case Definition 3 had a sensitivity of 97.3% (95% CI 95.7%-98.5%), a specificity of 76.6% (95% CI 74.3%-78.8%), a PPV of 63.9% (95% CI 61.6%-66.0%), and an NPV of 98.6% (95% CI 97.7%-99.1%; Supplemental Appendix S2).

**Figure 2:**
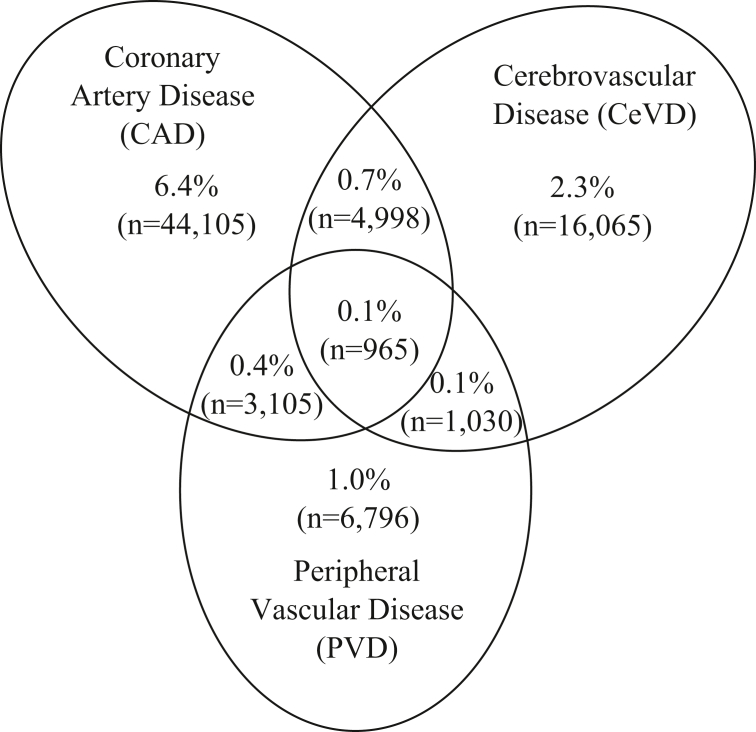
Venn diagram demonstrating proportion of active patients in the Canadian Primary Care Sentinel Surveillance Network captured using the strongest cardiovascular disease case definitions and the overlap of coronary artery disease (CAD), cerebrovascular disease (CeVD), and peripheral vascular disease (PVD) diagnosis in primary care settings (n = 689,301).

A total of 689,301 active patients were in the CPCSSN dataset. In comparison to those in the Canadian Census, CPCSSN patients were more likely to be female (56.3% vs 50.7%) and older (52 years vs 42 years). Application of Case Definition 2 to the active CPCSSN patient population (n = 689,301) resulted in an estimated prevalence of CVD among patients seen in primary care settings of 11.2% (95% CI 11.1%-11.3%; n = 77,064). When this is separated by type of CVD, 7.7% of patients (n = 53,173) had CAD, 3.4% (n = 23,058) had CeVD, and 1.7% (n = 11,896) had PVD. Case Definition 1 provided a similar prevalence estimate (9.6%, 95% CI 9.5%-9.6%). As expected, Case Definitions 3 and 4 provided a much higher prevalence estimate (51.0%, 95% CI 50.9%-51.2% and 31.1%, 95% CI 31.0%-31.2%, respectively). Case Definitions 3 and 4 represent a population with a larger group of CVD-related conditions. Figure 2 describes the proportion and type of CVD identified among active CPCSSN patients, along with the prevalence of various combinations. The majority had CAD (6.4%), followed by CeVD (2.3%), PVD (1.0%), and both CAD and CeVD (0.7%; Fig. 2).

Table 3 characterized patients captured with CVD Case Definition 2, as compared to patients not captured with CVD Case Definition 2. Patients captured with CVD Case Definition 2 were significantly more likely to be male (55.4% vs 42.3%, *P* = < 0.001) and older (70.5 years [SD 14.4] vs 50.0 years [SD 18.7], *P* = < 0.001). Patients captured with CVD Case Definition 2 were more likely to be diagnosed with hypertension (61.7% vs 24.3%, *P* = < 0.001), dyslipidemia (72.7% vs 34.6%, *P* = < 0.001), and/or HF (19.7% vs 2.3%, *P* = < 0.001). They were also more likely to be obese (8.4% vs 5.9%, *P* = < 0.001). Seventy-five percent of patients were captured with CVD Case Definition 2 were prescribed a CVD medication, compared to 24.7% of those not captured (< 0.0001; Table 3).

**Table 3:** Patients with at least 1 visit to a primary care provider participating in the Canadian Primary Care Sentinel Surveillance Network between January 1, 2017 and December 31, 2019 (N = 689,301)

Variable name	Patients without CVD n = 612,237	Patients with CVD (Case Definition 2) n = 77,064	*P*
Patient sex, male	258,657 (42.3)	42,644 (55.4)	< 0.0001
Patient age, mean (SD)	50.0 (18.7)	70.5 (14.4)	< 0.0001
Annual visit frequency, mean (SD)	2.6 (3.0)	4.8 (5.5)	< 0.0001
Systolic BP, mm Hg, mean (SD)	125 (16)	130 (17)	< 0.0001
Diastolic BP, mm Hg, mean (SD)	76.8 (10)	74 (11)	< 0.0001
Obesity (BMI ≥ 30)	35,965 (5.9)	6483 (8.4)	< 0.0001
Diabetes	68,515 (11.2)	23,169 (30.1)	< 0.0001
Hypertension	148,654 (24.3)	47,509 (61.7)	< 0.0001
Heart failure	14,003 (2.3)	15,187 (19.7)	< 0.0001
Dyslipidemia	211,529 (34.6)	55,992 (72.7)	< 0.0001
CVD medication	151,236 (24.7)	57,924 (75.2)	< 0.0001
Cardiac therapy	7935 (1.3)	18,917 (24.6)	< 0.0001
Beta-blockers	44,886 (7.3)	33,555 (43.5)	< 0.0001
Calcium-channel blockers	59,365 (9.7)	25,720 (33.4)	< 0.0001
ACE inhibitors	81,693 (13.3)	37,563 (48.7)	< 0.0001
ARB	47,782 (7.8)	17,544 (22.8)	< 0.0001

Values are n (%), unless otherwise indicated.

ACE, angiotensin-converting enzyme; ARB, angiotensin receptor blocker; BMI, body mass index; BP, blood pressure; CVD, cardiovascular disease; SD, standard deviation.

## Discussion

The creation and validation of an EMR-based case definition for CVD will be beneficial for disease surveillance as well as research and quality improvement. CVD Case Definition 2 had strong sensitivities, specificities, PPV, and NPV, compared to our reference data, reflecting satisfactory capture based on the available documentation. Our EMR-based CVD Case Definition 2 included CAD, CeVD, and PVD and suggests an overall prevalence of 11.2% within primary care settings, with some patients managed in primary care diagnosed with more than one of these conditions (ie, CAD, CeVD, and PVD). Further, among the patients captured with CVD Case Definition 2, we found an association with several expected risk factors and comorbidities.^11^, ^12^, ^13^, ^14^, ^15^

Application of our CVD, CAD, CeVD, and PVD definitions found prevalences of 11.2%, 7.7%, 3.7%, and 1.7%, respectively. Related literature has defined CVD subtypes, including acute myocardial infarction, ischemic heart disease, and CAD within similar ranges.^24^, ^25^, ^26^, ^27^, ^28^, ^29^, ^30^ An analysis from the Global Burden of Disease Study suggests that 9% of the population is diagnosed with ischemic heart disease, HF, or stroke.^42^

Much of the previous literature on CVD case validation has defined individual types of CVD and often has used administrative data that were not generalizable to a broad primary care population.^25^, ^26^, ^27^, ^28^, ^29^, ^30^, ^31^, ^32^, ^33^, ^34^ In fact, application of administrative case definitions in our study produced prevalences of 51.0% and 31.1%, respectively. Few studies have examined CVD overall, which is relevant to understanding the combined burden of atherosclerotic disease, which shares common risk factors and primary and secondary prevention strategies.^24^^,^^32^^,^^39^ Additionally, the existing literature is variable regarding application of case definitions to only an adult population or lack of requirement of an age modifier. Our CVD case definition, which included CAD, CeVD, and PVD, reported a sensitivity of 76.9% and a PPV of 75.4%. The strong sensitivity of our case definition highlights its potential application for understanding CVD epidemiology.^43^

We expect that our CVD case definition was impacted by the performance of our case definitions for CAD, CeVD, and PVD. In primary care, CAD is the most frequently diagnosed of these CVD types. Case Definition 15 had a sensitivity of 91.6%, and a PPV of 74.8%. Tu et al. used a linked dataset of administrative data and EMR-billing records to validate a definition for ischemic heart disease, reporting a lower sensitivity of 77.0%, and a similar PPV of 78.8%.^26^ The high sensitivity of the CAD definition can support studies aimed at inequalities in outcomes and epidemiology. The moderate PPV suggests that the CAD definition could be used cautiously to define cohorts for medication studies.

CeVD Case Definition 1 did not perform as well as the CAD definition—it had a sensitivity of 77.6%, a PPV of 65.0%, and an estimated prevalence of 3.4%. Tu et al. validated a definition for stroke/transient ischemic attack using administrative data and reported a sensitivity of 68.0% and a prevalence of 3.0%, which are similar to the values in our study.^25^ PVD had a low sensitivity of 36.6% and a PPV of 42.9% and should be used with caution. PVD is underdiagnosed and undertreated in primary care settings despite development of diagnostic tools and management guidelines.^37^ PVD often goes undiagnosed because the physical findings vary widely, and it can be difficult to diagnose because the symptoms may be more subtle.^36^ This PVD definition should be used in combination with CAD and CeVD definitions to capture patients with CVD.

Age and sex are important risk factors for CVD. Similar to previous literature, our case definition found a male predisposition for CVD and demonstrated strong associations with age, obesity, and hypertension.^3^, ^4^, ^5^, ^6^^,^^44^, ^45^, ^46^ Patients captured with our Case Definition 2 have characteristics similar to those reported in previous literature, lending credence to the accuracy and utility of our definition. As the Canadian population ages, an improved understanding of the epidemiology of CVD and its relationship to CVD risk factors can better inform and prepare for healthcare system needs.^43^^,^^45^ Application of a valid CVD case definition can inform disease epidemiology, which often highlights modifiable risk factors and has the potential to yield efficient utilization of time and resources by researchers and policy makers. Additionally, such application can streamline the disease surveillance mechanisms by characterizing the population at risk.^43^^,^^47^

In most healthcare systems in high-income countries, such as Canada, primary care providers are often the stewards of healthcare, as they are frequently the first point of contact for patients. Primary care providers are involved in primary prevention, diagnosis, treatment, and secondary prevention of diseases for their patients. In such settings, having a tool that facilitates improved disease surveillance can inform management algorithms and policies that are designed to manage the ever-rising volume of cases of CVD. 16, 17, 18, 19, 20, 21 As demonstrated in our study, patients captured by CVD Case Definition 2 visited their primary care provider more frequently each year, compared to patients not captured by CVD Case Definition 2. Visit frequency can be attributed to both CVD and multiple comorbidities and risk factors, such as hypertension and diabetes. Evidence has demonstrated that primary and secondary prevention using evidence-based interventions can reduce CVD mortality and morbidity.^16^, ^17^, ^18^, ^19^, ^20^, ^21^ This finding merits attention for primary care system design and resourcing aimed at both primary and secondary prevention of CVD.^3^, ^4^, ^5^, ^6^^,^^10^, ^11^, ^12^ Nevertheless, addressing these risk factors with evidence-based interventions in primary care is possible and is likely to have a greater impact on those with CVD.^3^, ^4^, ^5^, ^6^^,^^48^, ^49^, ^50^ Given that CVD impacts multiple organs (ie, brain, heart, peripheral vasculature), it is co-managed by multiple practitioners, again reinforcing the need for tools to support primary care providers, who often frequently support care coordination and navigation for patients with multiple comorbid conditions.^9^

### Limitations

Although this study validated a case definition for CVD based on primary care EMR data with reasonable face validity, it is not without several limitations. The CPCSSN dataset includes a large number of patients and providers from various practice types and locations across Canada, but we are uncertain whether our cohort is representative of other jurisdictions. Patients within the CPCSSN dataset were more likely to be female and older than those in the Canadian Census. The CPCSSN represents a population that had sought and accessed primary healthcare; future research should age- and sex-standardize prevalence estimates to describe prevalence estimates in a general Canadian population. This study accessed primary care EMR data for secondary purposes, but it was unable to assess the accuracy of the documentation. This means that in some instances, CVD or its component parts may be overdiagnosed or underdiagnosed by the healthcare provider. This possibility is less likely for serious conditions, such as CAD and CeVD, which often have symptoms that are noticeable, require specific care in community settings, and usually affect activities of daily living. Another possibility is that PVD diagnoses were omitted or underdiagnosed in some cases,^38^, ^39^, ^40^ especially given the fact that PVD is known to be underdiagnosed and is often managed outside of primary care—hence the less-robust agreement.^36^ A related issue is that in some cases the medical records assessed do not specify the type of CVD; therefore, using a broader CVD definition to describe patients may be more accurate, compared to capture of a type of CVD. Previous studies have demonstrated imperfect data quality of EMR records.^51^^,^^52^ We cannot account for specialist consultations or specific investigations and operative reports (ie, angiography, stress testing) that might be absent in primary care data, but present in administrative or hospital-based data. However, our focus on diagnosis of CVD in primary care settings informs secondary prevention strategies provided by primary care providers. Future studies aiming to describe the complete spectrum of care could link primary and tertiary care data for CVD, as well as assess unstructured data within a large and robust training set.

## Conclusions

This study describes a validated highly specific definition of CVD, which is comprised of CAD, CeVD, and PVD. The CVD cohort described has patient characteristics that include comorbid conditions and risk factors consistent with those reported in the literature. Understanding the prevalence and disease burden for patients with CVD who are being managed in primary care settings can improve care for this important disease.
