# Supplementary material for: Validation of a Case Definition to Identify Patients Diagnosed With Cardiovascular Disease in Canadian Primary Care Practices
Source: CJC Open. 2023 Apr 22;5(7):567–76. doi: 10.1016/j.cjco.2023.04.003 (PMC10366639; doi:10.1016/j.cjco.2023.04.003)
Supplement: Supplementary Material [file mmc1.docx]

**Supplemental Appendix S1**: Data extraction table

| Patient ID | Diagnosis name | CVD (Y/N) | Type of CVD (CAD, CeVD, PVD, unknown) | Key words related to CVD | Related conditions | CVD symptoms listed | Medication name |
| --- | --- | --- | --- | --- | --- | --- | --- |
|  |  |  |  |  |  |  |  |
|  |  |  |  |  |  |  |  |
|  |  |  |  |  |  |  |  |

**Supplemental Appendix S2**: Case Definitions for Coronary Artery Disease (CAD), Cerebrovascular Disease (CeVD) and Peripheral Vascular Disease (PVD). Together these case definitions informed case definitions for cardiovascular disease (CVD).

**CVD-CAD Case Validation**

| CAD case definition 1 | Patients aged 19 years or older  AND  >=2 health condition, billing, or encounter diagnosis for ICD9 410-414 within 1 year |
| --- | --- |
| CAD case definition 2 | Patients aged 19 years or older  AND  >=2 health condition, billing, or encounter diagnosis for ICD9 410-414 within 2 year |
| CAD case definition 3 | Patients aged 19 years or older  AND  >=2 health condition, billing, or encounter diagnosis for ICD9 410-414 within 2 year  AND  >=2 prescriptions for: ATC codes: C01DA02, C01DA05, C01DA08, C01DA14), other cardiac drugs (ATC codes: C01EB09), beta blocking agents (ATC codes: C07AA02, C07AA03, C07AA05, C07AA06, C07AA12, C07AB02, C07AB03, C07AB04, C07AB07, C07AG01, C07BA05, C07BA06, C07BA12, C07CA03, C07CB03), calcium channel blockers (ATC codes: C08CA01, C08CA02, C08CA04, C08CA05, C08CA06, C08DA01, C08DB01), angiotensin converting enzyme inhibitors (ACEI ATC codes: C09AA01, C09AA02, C09AA03, C09AA04, C09AA05, C09AA06, C09AA07, C09AA08, C09AA09, C09AA10, C09BA02, C09BA03, C09BA04, C09BA06, C09BA08), angiotensin II antagonists (ATC codes: C09CA01, C09CA02, C09CA03, C09CA04, C09CA06, C09CA07, C09DA01, C09DA02, C09DA03, C09DA04, C09DA06, C09DA07) in 3 years. |
| CAD case definition 4 | Patients aged 19 years or older  AND  >=2 health condition, billing, or encounter diagnosis for ICD9 410-414 within 2 year  OR  >=2 prescriptions for: ATC codes: C01DA02, C01DA05, C01DA08, C01DA14), other cardiac drugs (ATC codes: C01EB09), beta blocking agents (ATC codes: C07AA02, C07AA03, C07AA05, C07AA06, C07AA12, C07AB02, C07AB03, C07AB04, C07AB07, C07AG01, C07BA05, C07BA06, C07BA12, C07CA03, C07CB03), calcium channel blockers (ATC codes: C08CA01, C08CA02, C08CA04, C08CA05, C08CA06, C08DA01, C08DB01), angiotensin converting enzyme inhibitors (ACEI ATC codes: C09AA01, C09AA02, C09AA03, C09AA04, C09AA05, C09AA06, C09AA07, C09AA08, C09AA09, C09AA10, C09BA02, C09BA03, C09BA04, C09BA06, C09BA08), angiotensin II antagonists (ATC codes: C09CA01, C09CA02, C09CA03, C09CA04, C09CA06, C09CA07, C09DA01, C09DA02, C09DA03, C09DA04, C09DA06, C09DA07) in 3 years. |
| CAD case definition 5 | >=2 health condition, billing, or encounter diagnosis for ICD-Codes 410.XX, 412.XX and 413.XX within 1 year |
| CAD case definition 6 | >=2 health condition, billing, or encounter diagnosis for ICD-Codes 410.XX, 412.XX and 413.XX within 1 year  AND  >=2 prescriptions for: ATC codes: C01DA02, C01DA05, C01DA08, C01DA14), other cardiac drugs (ATC codes: C01EB09), beta blocking agents (ATC codes: C07AA02, C07AA03, C07AA05, C07AA06, C07AA12, C07AB02, C07AB03, C07AB04, C07AB07, C07AG01, C07BA05, C07BA06, C07BA12, C07CA03, C07CB03), calcium channel blockers (ATC codes: C08CA01, C08CA02, C08CA04, C08CA05, C08CA06, C08DA01, C08DB01), angiotensin converting enzyme inhibitors (ACEI ATC codes: C09AA01, C09AA02, C09AA03, C09AA04, C09AA05, C09AA06, C09AA07, C09AA08, C09AA09, C09AA10, C09BA02, C09BA03, C09BA04, C09BA06, C09BA08), angiotensin II antagonists (ATC codes: C09CA01, C09CA02, C09CA03, C09CA04, C09CA06, C09CA07, C09DA01, C09DA02, C09DA03, C09DA04, C09DA06, C09DA07) |
| CAD case definition 7 | >=2 health condition, billing, or encounter diagnosis for ICD-Codes 410.XX, 412.XX and 413.XX within 1 year  OR  >=2 prescriptions for: ATC codes: C01DA02, C01DA05, C01DA08, C01DA14), other cardiac drugs (ATC codes: C01EB09), beta blocking agents (ATC codes: C07AA02, C07AA03, C07AA05, C07AA06, C07AA12, C07AB02, C07AB03, C07AB04, C07AB07, C07AG01, C07BA05, C07BA06, C07BA12, C07CA03, C07CB03), calcium channel blockers (ATC codes: C08CA01, C08CA02, C08CA04, C08CA05, C08CA06, C08DA01, C08DB01), angiotensin converting enzyme inhibitors (ACEI ATC codes: C09AA01, C09AA02, C09AA03, C09AA04, C09AA05, C09AA06, C09AA07, C09AA08, C09AA09, C09AA10, C09BA02, C09BA03, C09BA04, C09BA06, C09BA08), angiotensin II antagonists (ATC codes: C09CA01, C09CA02, C09CA03, C09CA04, C09CA06, C09CA07, C09DA01, C09DA02, C09DA03, C09DA04, C09DA06, C09DA07) |
| CAD case definition 8 | >=2 health condition, billing, or encounter diagnosis for ICD-Codes 410.XX, 412.XX and 413.XX within 2 years |
| CAD case definition 9 | >=2 health condition, billing, or encounter diagnosis for ICD-Codes 410.XX, 412.XX and 413.XX within 3 years |
| CAD case definition 10 | >=2 health condition, billing, or encounter diagnosis for ICD-Codes 410.XX, 412.XX and 413.XX within 3 year  AND  >=2 prescriptions for ATC codes: C01DA02, C01DA05, C01DA08, C01DA14), other cardiac drugs (ATC codes: C01EB09), beta blocking agents (ATC codes: C07AA02, C07AA03, C07AA05, C07AA06, C07AA12, C07AB02, C07AB03, C07AB04, C07AB07, C07AG01, C07BA05, C07BA06, C07BA12, C07CA03, C07CB03), calcium channel blockers (ATC codes: C08CA01, C08CA02, C08CA04, C08CA05, C08CA06, C08DA01, C08DB01), angiotensin converting enzyme inhibitors (ACEI ATC codes: C09AA01, C09AA02, C09AA03, C09AA04, C09AA05, C09AA06, C09AA07, C09AA08, C09AA09, C09AA10, C09BA02, C09BA03, C09BA04, C09BA06, C09BA08), angiotensin II antagonists (ATC codes: C09CA01, C09CA02, C09CA03, C09CA04, C09CA06, C09CA07, C09DA01, C09DA02, C09DA03, C09DA04, C09DA06, C09DA07) |
| CAD case definition 11 | >=2 health condition, billing, or encounter diagnosis for ICD-Codes 410.XX, 412.XX and 413.XX within 3 year  OR  >=2 prescriptions for ATC codes: C01DA02, C01DA05, C01DA08, C01DA14), other cardiac drugs (ATC codes: C01EB09), beta blocking agents (ATC codes: C07AA02, C07AA03, C07AA05, C07AA06, C07AA12, C07AB02, C07AB03, C07AB04, C07AB07, C07AG01, C07BA05, C07BA06, C07BA12, C07CA03, C07CB03), calcium channel blockers (ATC codes: C08CA01, C08CA02, C08CA04, C08CA05, C08CA06, C08DA01, C08DB01), angiotensin converting enzyme inhibitors (ACEI ATC codes: C09AA01, C09AA02, C09AA03, C09AA04, C09AA05, C09AA06, C09AA07, C09AA08, C09AA09, C09AA10, C09BA02, C09BA03, C09BA04, C09BA06, C09BA08), angiotensin II antagonists (ATC codes: C09CA01, C09CA02, C09CA03, C09CA04, C09CA06, C09CA07, C09DA01, C09DA02, C09DA03, C09DA04, C09DA06, C09DA07) |
| CAD case definition 12 | Patients aged 19 years or older  AND  >=2 health condition, billing, or encounter diagnosis for ICD9 410-414 within 3 year |
| CAD case definition 13 | >=1 health condition, billing, or encounter diagnosis for ICD9 410-414  AND  >=2 prescriptions for ATC codes: C01DA02, C01DA05, C01DA08, C01DA14), other cardiac drugs (ATC codes: C01EB09), beta blocking agents (ATC codes: C07AA02, C07AA03, C07AA05, C07AA06, C07AA12, C07AB02, C07AB03, C07AB04, C07AB07, C07AG01, C07BA05, C07BA06, C07BA12, C07CA03, C07CB03), calcium channel blockers (ATC codes: C08CA01, C08CA02, C08CA04, C08CA05, C08CA06, C08DA01, C08DB01), angiotensin converting enzyme inhibitors (ACEI ATC codes: C09AA01, C09AA02, C09AA03, C09AA04, C09AA05, C09AA06, C09AA07, C09AA08, C09AA09, C09AA10, C09BA02, C09BA03, C09BA04, C09BA06, C09BA08), angiotensin II antagonists (ATC codes: C09CA01, C09CA02, C09CA03, C09CA04, C09CA06, C09CA07, C09DA01, C09DA02, C09DA03, C09DA04, C09DA06, C09DA07) |
| CAD case definition 14 | >=2 health condition, billing, or encounter diagnosis for ICD9 410-414  AND  >=2 prescriptions for ATC codes: C01DA02, C01DA05, C01DA08, C01DA14), other cardiac drugs (ATC codes: C01EB09), beta blocking agents (ATC codes: C07AA02, C07AA03, C07AA05, C07AA06, C07AA12, C07AB02, C07AB03, C07AB04, C07AB07, C07AG01, C07BA05, C07BA06, C07BA12, C07CA03, C07CB03), calcium channel blockers (ATC codes: C08CA01, C08CA02, C08CA04, C08CA05, C08CA06, C08DA01, C08DB01), angiotensin converting enzyme inhibitors (ACEI ATC codes: C09AA01, C09AA02, C09AA03, C09AA04, C09AA05, C09AA06, C09AA07, C09AA08, C09AA09, C09AA10, C09BA02, C09BA03, C09BA04, C09BA06, C09BA08), angiotensin II antagonists (ATC codes: C09CA01, C09CA02, C09CA03, C09CA04, C09CA06, C09CA07, C09DA01, C09DA02, C09DA03, C09DA04, C09DA06, C09DA07) |
| CAD case definition 15 | >=1 health condition, billing, or encounter diagnosis for ICD9 410-414 |
| CAD case definition 16 | >=2 health condition, billing, or encounter diagnosis for ICD9 410-414 |
| CAD case definition 17 | >=1 health condition, billing, or encounter diagnosis for ICD9 410-414  AND  >=1 prescriptions for ATC codes: C01DA02, C01DA05, C01DA08, C01DA14), other cardiac drugs (ATC codes: C01EB09), beta blocking agents (ATC codes: C07AA02, C07AA03, C07AA05, C07AA06, C07AA12, C07AB02, C07AB03, C07AB04, C07AB07, C07AG01, C07BA05, C07BA06, C07BA12, C07CA03, C07CB03), calcium channel blockers (ATC codes: C08CA01, C08CA02, C08CA04, C08CA05, C08CA06, C08DA01, C08DB01), angiotensin converting enzyme inhibitors (ACEI ATC codes: C09AA01, C09AA02, C09AA03, C09AA04, C09AA05, C09AA06, C09AA07, C09AA08, C09AA09, C09AA10, C09BA02, C09BA03, C09BA04, C09BA06, C09BA08), angiotensin II antagonists (ATC codes: C09CA01, C09CA02, C09CA03, C09CA04, C09CA06, C09CA07, C09DA01, C09DA02, C09DA03, C09DA04, C09DA06, C09DA07) |
| CAD case definition 18 | >=1 health condition for ICD9 410-414  OR  >=2 billing or encounter diagnosis for ICD9 410-414 |
| CAD case definition 19 | >=1 health condition for ICD9 410-414  OR  >=2 billing or encounter diagnosis for ICD9 410-414  AND  >=2 prescriptions for ATC codes: C01DA02, C01DA05, C01DA08, C01DA14), other cardiac drugs (ATC codes: C01EB09), beta blocking agents (ATC codes: C07AA02, C07AA03, C07AA05, C07AA06, C07AA12, C07AB02, C07AB03, C07AB04, C07AB07, C07AG01, C07BA05, C07BA06, C07BA12, C07CA03, C07CB03), calcium channel blockers (ATC codes: C08CA01, C08CA02, C08CA04, C08CA05, C08CA06, C08DA01, C08DB01), angiotensin converting enzyme inhibitors (ACEI ATC codes: C09AA01, C09AA02, C09AA03, C09AA04, C09AA05, C09AA06, C09AA07, C09AA08, C09AA09, C09AA10, C09BA02, C09BA03, C09BA04, C09BA06, C09BA08), angiotensin II antagonists (ATC codes: C09CA01, C09CA02, C09CA03, C09CA04, C09CA06, C09CA07, C09DA01, C09DA02, C09DA03, C09DA04, C09DA06, C09DA07) |
| CAD case definition 20 | >=1 health condition for ICD9 410-414  OR  >=2 billing or encounter diagnosis for ICD9 410-414  AND  >=1 prescriptions for ATC codes: C01DA02, C01DA05, C01DA08, C01DA14), other cardiac drugs (ATC codes: C01EB09), beta blocking agents (ATC codes: C07AA02, C07AA03, C07AA05, C07AA06, C07AA12, C07AB02, C07AB03, C07AB04, C07AB07, C07AG01, C07BA05, C07BA06, C07BA12, C07CA03, C07CB03), calcium channel blockers (ATC codes: C08CA01, C08CA02, C08CA04, C08CA05, C08CA06, C08DA01, C08DB01), angiotensin converting enzyme inhibitors (ACEI ATC codes: C09AA01, C09AA02, C09AA03, C09AA04, C09AA05, C09AA06, C09AA07, C09AA08, C09AA09, C09AA10, C09BA02, C09BA03, C09BA04, C09BA06, C09BA08), angiotensin II antagonists (ATC codes: C09CA01, C09CA02, C09CA03, C09CA04, C09CA06, C09CA07, C09DA01, C09DA02, C09DA03, C09DA04, C09DA06, C09DA07) |

| Agreement between National Reference set for CVD-CAD and case definitions  N=2017 | | | | | |
| --- | --- | --- | --- | --- | --- |
|  | Sen | Spec | PPV | NPV | Accuracy |
| CAD case definition 1 | 42.99 (33.46-52.92) | 99.27 (98.77-99.6) | 76.67 (65.11-85.26) | 96.88 (96.35-97.34) | 96.28 (95.36-97.06) |
| CAD case definition 2 | 43.93 (34.34-53.85) | 99.27 (98.77-99.6) | 77.05 (65.63-85.51) | 96.93 (96.39-97.39) | 96.33 (95.42-97.11) |
| CAD case definition 3 | 34.58 (25.65-44.39) | 99.53 (99.11-99.78) | 80.43 (67.08-89.24) | 96.45 (95.95-96.89) | 96.08 (95.14-96.89) |
| CAD case definition 4 | 85.05 (76.86-91.2) | 81.15 (79.32-82.88) | 20.18 (18.28-22.22) | 98.98 (98.4-99.35) | 81.36 (79.59-83.04) |
| CAD case definition 5 | 16.82 (10.29-25.28) | 99.58 (99.18-99.82) | 69.23 (50.03-83.49) | 95.53 (95.15-95.88) | 95.19 (94.16-96.08) |
| CAD case definition 6 | 14.02 (8.06-22.07) | 99.74 (99.39-99.91) | 75.0 (52.63-89.01) | 95.39 (95.04-95.72) | 95.19 (94.16-96.08) |
| CAD case definition 7 | 78.5 (69.51-85.86) | 81.26 (79.43-82.98) | 19.0 (17.0-21.19) | 98.54 (97.91-98.98) | 81.11 (79.33-82.8) |
| CAD case definition 8 | 16.82 (10.29-25.28) | 99.58 (99.18-99.82) | 69.23 (50.03-83.49) | 95.53 (95.15-95.88) | 95.19 (94.16-96.08) |
| CAD case definition 9 | 16.82 (10.29-25.28) | 99.58 (99.18-99.82) | 69.23 (50.03-83.49) | 95.53 (95.15-95.88) | 95.19 (94.16-96.08) |
| CAD case definition 10 | 14.02 (8.06-22.07) | 99.74 (99.39-99.91) | 75.00 (52.63-89.01) | 95.39 (95.04-95.72) | 95.19 (94.16-96.08) |
| CAD case definition 11 | 78.5 (69.51-85.86) | 81.26 (79.43-82.98) | 19.0 (17.0-21.19) | 98.54 (97.91-98.98) | 81.11 (79.33-82.8) |
| CAD case definition 12 | 43.93 (34.34-53.85) | 99.27 (98.77-99.6) | 77.05 (65.63-85.51) | 96.93 (96.39-97.39) | 96.33 (95.42-97.11) |
| CAD case definition 13 | 71.96 (62.45-80.22) | 99.11 (98.58-99.48) | 81.91 (73.55-88.06) | 98.44 (97.9-98.84) | 97.67 (96.91-98.28) |
| CAD case definition 14 | 42.06 (32.58-51.99) | 99.53 (99.11-99.78) | 83.33 (71.52-90.87) | 96.84 (96.31-97.3) | 96.48 (95.58-97.24) |
| CAD case definition 15 | 91.59 (84.63-96.08) | 98.27 (97.58-98.8`) | 74.81 (67.82-80.71) | 99.52 (99.11-99.74) | 97.92 (97.2-98.5) |
| CAD case definition 16 | 51.4 (41.54-61.18) | 99.16 (98.64-99.52) | 77.46 (67.11-85.27) | 97.33 (96.77-97.79) | 96.63 (95.75-97.37) |
| CAD case definition 17 | 76.64 (67.47-84.27) | 98.85 (98.26-99.28) | 78.85 (70.83-85.12) | 98.69 (98.17-99.07) | 97.67 (96.91-98.28) |
| CAD case definition 18 | 79.44 (70.54-86.64) | 98.9 (98.32-99.32) | 80.19 (72.35-86.23) | 98.85 (98.34-99.2) | 97.87 (97.14-98.45) |
| CAD case definition 19 | 64.49 (54.65-73.5) | 99.37 (98.91-99.67) | 85.19 (76.28-91.14) | 98.04 (97.48-98.47) | 97.52 (96.74-98.15) |
| CAD case definition 20 | 68.22 (58.52-76.89) | 99.27 (98.77-99.6) | 83.91 (75.28-89.93) | 98.24 (97.69-98.66) | 97.62 (96.86-98.24) |

**CVD-CeVD Case Validation**

| CeVD Case definitions | |
| --- | --- |
| CeVD case definition 1 | Patients aged 19 years or older  AND  >=1 health condition, billing, or encounter diagnosis for ICD9 430-438 |
| CeVD case definition 2 | Patients aged 19 years or older  AND  >=1 health condition, billing, or encounter diagnosis for ICD9 430-438 within 5 years |
| CeVD case definition 3 | >=1 health condition, billing, or encounter diagnosis for ICD9 362.3, 430,431,434.x, 436, 435.x |
| CeVD case definition 4 | >=2 health condition, billing, or encounter diagnosis for ICD9 362.3, 430,431,434.x, 436, 435.x within 2 years |
| CeVD case definition 5 | >=1 health condition for ICD9 430-438  OR  >= 2 billing, or encounter diagnosis for ICD9 430-438 |

| Agreement between National Reference set and CeVD case definitions  N=2017 | | | | | |
| --- | --- | --- | --- | --- | --- |
|  | Sen | Spec | PPV | NPV | Accuracy |
| CeVD case definition 1 | 77.61 (65.78-86.89) | 98.56 (97.93-99.04) | 65.0 (55.71-73.27) | 99.23 (98.8-99.5) | 97.87 (97.14-98.45) |
| CeVD case definition 2 | 50.75 (38.24-63.18) | 99.23 (98.73-99.57) | 69.39 (56.51-79.82) | 98.32 (97.87-98.68) | 97.62 (96.86-98.24) |
| CeVD case definition 3 | 23.88(14.31-35.86) | 99.64 (99.26-99.86) | 69.57 (49.31-84.3) | 97.44 (97.09-97.76) | 97.12 (96.3-97.81) |
| CeVD case definition 4 | 23.88(14.31-35.86) | 99.64 (99.26-99.86) | 69.57 (49.31-84.3) | 97.44 (97.09-97.76) | 97.12 (96.3-97.81) |
| CeVD case definition 5 | 64.18 (51.53-75.53) | 99.08 (98.55-99.45) | 70.49 (59.33-79.64) | 98.77 (98.32-99.11) | 97.92 (97.2-98.5) |

**CVD-PVD Case Definition Validation**

| PVD Case Definitions | |
| --- | --- |
| PVD Case Definition 1 | >=1 health condition, billing, or encounter diagnosis for ICD9 440.xx, 443.xx |
| PVD Case Definition 2 | >=2 health condition, billing, or encounter diagnosis for ICD9 440.xx, 443.xx |

| Agreement between National Reference set for CVD-Peripheral Vascular and case definitions  N=2017 | | | | | |
| --- | --- | --- | --- | --- | --- |
|  | Sen | Spec | PPV | NPV | Accuracy |
| PVD Case Definition 1 | 36.59 (22.12-53.06) | 98.99 (98.44-99.38) | 42.86 (29.29-57.59) | 98.69 (98.35-98.96) | 97.72 (96.97-98.33) |
| PVD Case Definition 2 | 12.2 (4.08-26.2) | 99.75 (99.41-99.92) | 50.0 (23.14-76.86) | 98.21 (97.99-98.4) | 97.97 (97.25-98.54) |

**CVD Case Definitions**

| Cardiovascular Disease Case Definitions | |
| --- | --- |
| Case definition 1 | CAD: ≥1 HC, billing or encounter dx for ICD-9 410-414 AND ≥2 medications for ATC codes starting with C01, C07, C08, C09  **OR**  CeVD: ≥1 HC, billing or encounter dx for ICD-9 430-438  **OR**  PVD: ≥1 HC, billing or encounter dx for ICD-9 440.xx, or 443.xx |
| Case definition 2 | CAD: ≥1 HC, billing or encounter dx for ICD-9 410-414  **OR**  CeVD: ≥1 HC, billing or encounter dx for ICD-9 430-438  **OR**  PVD: ≥1 HC, billing or encounter dx for ICD-9 440.xx, or 443.xx |
| Case definition 3 | ≥1 HC, billing or encounter dx for ICD-9 390-429, 430-448,458  **OR**  ≥1 ATC code from medication table for B01A, C01A, C01B, C01CA17, C01D, C02AA, C02AB, C02C, C02D, C02L, C03, C04AD, C05BA, C07AA01, C07AA02 C07AA03, C07AA04, C07AA06, C07AA07, C07AA12, C07AB, C07AG, C07B, C07C, C08, C09, C10 |
| Case definition 4 | ≥1 HC, billing or encounter dx within 1 year for ICD-9 390-429, 430-448,458  **OR**  ≥1 ATC code from medication table within 1 year for B01A, C01A, C01B, C01CA17, C01D, C02AA, C02AB, C02C, C02D, C02L, C03, C04AD, C05BA, C07AA01, C07AA02, C07AA03, C07AA04, C07AA06, C07AA07, C07AA12, C07AB, C07AG, C07B, C07C, C08, C09, C10 |

| Agreement between National Reference set and CVD case definitions  N=2017 | | | | | |
| --- | --- | --- | --- | --- | --- |
|  | Sen | Spec | PPV | NPV | Accuracy |
| CVD case definition 1 | 68.47 (61.6-74.8) | 97.79 (97.01-98.42) | 77.65 (71.61-82.72) | 96.52 (95.77-97.14) | 94.84 (93.79-95.77) |
| CVD case definition 2 | 76.85 (70.43-82.46) | 97.19 (96.32-97.9) | 75.36 (69.79-80.2) | 97.4 (96.69-97.97) | 95.14 (94.11-96.04) |
| CVD case definition 3 | 94.58 (90.51-97.26) | 60.09 (57.79-62.35) | 20.96 (19.9-22.06) | 99.0 (98.24-99.44) | 63.56 (61.42-65.66) |
| CVD case definition 4 | 59.11 (52.01-65.94) | 78.17 (76.2-80.05) | 23.26 (20.79-25.92) | 94.47 (93.53-95.28) | 76.25 (74.33-78.09) |

| Agreement between National Reference set for CVD, hypertension, CHF, NVAF and case definitions  N=2,357 | | | | | |
| --- | --- | --- | --- | --- | --- |
|  | Sen | Spec | PPV | NPV | Accuracy |
| CVD case definition 1 | 26.79 (23.29-30.52) | 98.73 (98.0-99.24) | 89.94 (84.73-93.52) | 76.06 (75.16-76.94) | 77.29 (75.4-79.11) |
| CVD case definition 2 | 29.95 (26.31-33.79) | 98.09 (97.24-98.74) | 86.96 (81.82-90.81) | 76.74 (75.78-77.67) | 77.79 (75.91-79.59) |
| CVD case definition 3 | **97.34 (95.7-98.47)** | **76.62 (74.33-78.81)** | **63.86 (61.64-66.03)** | **98.55 (97.66-99.1)** | **82.8 (81.08-84.42)** |
| CVD case definition 4 | 65.39 (61.44-69.19) | 91.31 (89.72-92.73) | 76.16 (72.77-79.25) | 86.14 (84.76-87.42) | 83.59 (81.9-85.18) |

21 Wei, W.-Q., Teixeira, P.L., Mo, H., Cronin, R.M., Warner, J.L. and Denny, J.C. (2016) ‘Combining billing codes, clinical notes, and medications from electronic health records provides superior phenotyping performance’, *Journal of the American Medical Informatics Association,* 23, pp. 20–27. Available at: <https://doi.org/10.1093/jamia/ocv130> (Accessed 29 June 2021).

27 Tu K, Wang M, Young J, Green D, Ivers N, Butt D, Jaakkimainen L, Kapral M. Validity of administrative data for identifying patients who have had a stroke or transient attach using EMERALD as a reference standard. Canadian Journal of Cardiology. 2013;29:1388-1394.

28 Tu K, Mitiku T, Lee D, Guo H, Tu J. Validation of physician billing and hospitalization data to identify patients with ischemic heart disease using data from the electronic medical record administrative data linked database (EMRALD). Can J Cardiol. 2010;26(7):e225-e228.

29 Ammann EM, Schweizer ML, Robinson HG, Eschol JO, Kafa R, Girotra S, Winiecki S, et al. Chart validation of inpatient ICD-9-CM administrative diagnosis codes for acute myocardial infraction (AMI) among intravenous immune globulin (IGIV) users in the sentinel distributed database. Pharmacoepidemiol Drug Saf. 2018;27(4):398-404. Doi:101002/pds.4398

30 Ahmad FS, Chan C, Rosenman MB, Post WS, Fort DG, Greenland P, Liu KJ, et al. Validity of Cardiovascular data from electronic sources: The multi-ethnic study of antherosclerosis and HealthLNK. Circulation. 2017;136(13):1207-1216. doi:10.1161/CIRCULATIONAHA.117.027436.

31 Goyal A, Norton CR, Thomas TN, Davis RL, Butler J, Ashok V, Zhao L, et al. Predictors of incident heart failure in a large insured population: A one million person-year follow-up study. Circ Heart Fail. 2010; 3:698-705. DOI: 10.1161/CIRCHEARTFAILURE.110.938175

32 Kivimaki M, Batty GD, Singh-Manoux A, Britton A, Brunner E, Shipley M. Validity of cardiovascular disease event ascertainment using linkage to UK hospital records. Epidemiology;2017:28(5):735-739. doi: 10.1097/EDE.0000000000000688

33 Finlayson G, Ekuma O, Yogendran M, Burland E, Forget E. The Additional Cost of Chronic Disease in Manitoba. Winnipeg, MB: Manitoba Centre for Health Policy, 2010.

34 Lix L, Sobhan S, St-Jean A, Daigle JM, Fisher A, Yu OH. Et al. Validity of an algorithm to identify cardiovascular deaths from administrative health records: A multi-database population-based cohort study. BMC Health Services Research. 2021;21:758. <https://doi.org/10.1186/s12913-021-06762-0>

35 Katz A, Martens P, Chateau D, Bogdanovic B, Koseva I, McDougall C, Boriskewich E. Understanding the Health System Use of Ambulatory Care Patients. Winnipeg, MB: Manitoba Centre for Health Policy, 2013.

36 Chartier M, Dart A, Tangri N, Komenda P, Walld R, Bogdanovic B, Burchill C, Koseva I, McGowan K-L, Rajotte L. Care of Manitobans Living with Chronic Kidney Disease. Winnipeg, MB: Manitoba Centre for Health Policy, 2015.
